# Characteristics of dry eye in patients with pre-existing Sjögren's syndrome according to the revised 2016 ACR-EULAR classification criteria

**DOI:** 10.1097/MD.0000000000014641

**Published:** 2019-03-01

**Authors:** Hyeon Jeong Yoon, Won Choi, Jee Myung Yang, Yong Sok Ji, Shin-Seok Lee, Kyung Chul Yoon

**Affiliations:** a Department of Ophthalmology, Chonnam National University Medical School and Hospital, Gwangju; b Graduate School of Medical Science and Engineering, Korea Advanced Institute of Science and Technology, Daejeon; c Department of Rheumatology, Chonnam National University Medical School and Hospital, Gwangju, Korea.

**Keywords:** classification criteria, dry eye, Sjögren's syndrome

## Abstract

To compare the characteristics of dry eye (DE) patients who did and did not satisfy the 2016 American College of Rheumatology (ACR)-European League Against Rheumatism (EULAR) classification criteria for primary Sjögren's syndrome (SS) among patients with pre-existing SS diagnosed according to the 2012 ACR criteria

This cross-sectional study evaluated 91 patients with pre-existing SS and 55 with non-SS DE. Patients with SS were divided into 2 groups according to whether they met the revised 2016 ACR-EULAR classification criteria for primary SS. Group 1 (n = 71) was comprised of patients who satisfied the revised 2016 criteria and group 2 (n = 20) was comprised of patients who did not satisfy the newly revised criteria. Group 3 consisted of 55 patients with non-SS DE. The ocular surface disease index (OSDI) score, tear break-up time (TBUT), Schirmer score, tear clearance rate (TCR), and corneal and conjunctival staining scores were evaluated and compared between the groups. Laboratory profiles, including antinuclear antibodies, rheumatoid factor levels, erythrocyte sedimentation rate, and C-reactive protein levels, and focus scores were analyzed.

TBUT, Schirmer, and corneal/conjunctival staining scores were significantly worse in both groups of patients with SS (groups 1 and 2) than in those with non-SS DE (group 3). However, there were no significant differences between groups 1 and 2 in laboratory findings as well as in ocular surface findings, including OSDI, TBUT, Schirmer score, TCR, and corneal/conjunctival staining scores. The focus score, which shows the level of lymphocytic infiltration in the salivary glands, was higher in group 1 than in group.

Of the patients with pre-existing SS who were diagnosed according to the 2012 ACR classification, patients who did not satisfy the 2016 ACR-EULAR classification criteria for primary SS showed similar ocular surface parameters and laboratory findings to patients who did meet the revised classification, except for focus score. There is no need to change the direction of treatment of DE in patients with pre-existing SS who did not meet the revised 2016 ACR-EULAR criteria.

## 1 Introduction

Sjögren's syndrome (SS), a chronic autoimmune disease mainly affecting the lacrimal and salivary glands, is associated with moderate to severe dry eye (DE) and dry mouth. Infiltration of lymphocytes into the exocrine glands, such as the sebaceous, sweat, salivary, and lacrimal glands, causes systemic multi-organ manifestations.^[1–4]^ In patients with SS, lacrimal glands are considered to be the primary targets of auto-immune attack, leading to aqueous tear-deficient DE.^[2,5,6]^ However, meibomian gland dysfunction may be involved as well, leading to evaporative DE.^[2,7–9]^

The signs and symptoms of SS-DE are similar to those of non-SS-DE. However, DE associated with SS generally has more severe clinical manifestations and ocular surface parameters compared to non-SS-DE.^[7,10–12]^ In addition, SS-DE develops at a younger age and progresses more severely and rapidly.^[2,13]^ Therefore, it often requires more potent steroids, immunomodulatory drugs, and autologous serum.^[6,14–17]^

Recently, the criteria for diagnosis of primary SS have changed. The 2012 American College of Rheumatology (ACR) classification criteria for diagnosis of SS included the following:

(1) positive serum anti-SSA/Ro and/or anti-SSB/La antibodies (or positive rheumatoid factor [RF] and antinuclear antibody [ANA] titers ≥1:320);
(2) labial salivary gland biopsy exhibiting focal lymphocytic sialadenitis with a focus score ≥1 per 4 mm^2^; and
(3) keratoconjunctivitis sicca with ocular staining score (OSS) ≥3.

Diagnosis of SS requires the presence of at least 2 of the 3 objective features.^[18]^ On the other hand, the revised 2016 classification criteria by the ACR-European League Against Rheumatism (EULAR) defines SS with a weighted score. It assigns 3 points each for positive salivary gland biopsy and positive anti-SSA antibodies, and 1 point each for unstimulated whole salivary flow ≤0.1 mL/min, Schirmer test result ≤5 mm/5 min, and OSS ≥5 or van Bijsterveld score ≥4. Diagnosis of SS requires a weighted score of 4.^[19,20]^

With respect to ophthalmologic assessment, Schirmer test results have been added to the revised criteria, and the threshold of OSS has been increased from 3 to 5. In addition, the immunologic profile in the revised criteria includes only anti-SSA/Ro antibodies, not ANA, RF, or anti-SSB/La antibodies.^[19,20]^ As a result, there are some patients among those diagnosed with SS according to the 2012 ACR classification criteria who no longer meet the criteria for diagnosis according to the revised 2016 ACR-EULAR classification.

In the field of rheumatology, there have been studies comparing systemic findings between the different classification criteria for primary SS.^[21–23]^ However, there have been no reports on differences in ocular findings between patients who did and did not satisfy the revised 2016 ACR-EULAR classification for primary SS. The purpose of this study was to compare the characteristics of patients who met the 2016 ACR-EULAR classification criteria for SS to patients with pre-existing SS as diagnosed by the 2012 ACR criteria from November 2014 to December 2015.

## 2 Methods

In this cross-sectional study, participants were recruited at the Department of Ophthalmology, Chonnam National University Hospital. Informed consent was obtained from each subject. Ethical approval was obtained from the Chonnam National University Hospital Institutional Review Board, and the study protocol followed the guidelines of the Declaration of Helsinki.

### 2.1 Study population

From November 2014 to December 2015, 91 patients (91 eyes) diagnosed with SS, and 55 age-and-sex matched patients (55 eyes) diagnosed with non-SS DE were recruited. DE inclusion criteria were: the presence of symptoms for >3 months, low tear break-up time (TBUT) ( ≤7 s), and low basal tear secretion (≤10 mm/5 min). Study exclusion criteria were:

(1) previous DE treatment other than artificial tears, including other eye drops or punctal plug insertion,
(2) taking systemic medications that could facilitate or inhibit tear production,
(3) history of contact lenses use, and
(4) history of ocular surgery or trauma.

Patients with pre-existing SS who satisfied the 2012 ACR classification criteria were divided into 2 groups according to whether they met the revised 2016 ACR-EULAR criteria for primary SS. Group 1 comprised of patients who satisfied the revised 2016 criteria, and group 2 comprised of patients who did not satisfy the newly revised criteria. Patients with non-SS-DE were placed in group 3.

### 2.2 Ocular surface parameters measurements

The ocular surface disease index (OSDI) score, TBUT, Schirmer score, tear clearance rate (TCR), and corneal/conjunctival staining scores were evaluated by the same investigator (KCY) at the first visit. Only “the worst” eye was assessed, and determined as follows:

(1) eye with the more severe conjunctival staining score, or
(2) the right eye in cases where conjunctival staining score in both eyes was the same.

The OSDI questionnaire was used to quantify vision-related quality of life and included the following subscales:

(1) ocular symptoms (OSDI symptoms),
(2) vision-related activities of daily living (OSDI visual function), and
(3) environmental triggers (OSDI trigger).

The total OSDI score and each subscale score, which ranged from 0 to 100, were analyzed.^[24,25]^

TBUT was assessed using a moistened fluorescein strip (Haag-Streit, Koeniz, Switzerland) and the time interval between the last complete blinking and the first appearance of a dry spot or disruption of the tear film was recorded in seconds. The examination was repeated 3 times, and the mean time was used for analysis. Schirmer score was measured using a calibrated sterile strip (Color Bar Schirmer Tear Test; Eagle Vision Inc., Memphis) with topical anesthesia (0.5% proparacaine chloride). The sterile strips were placed in the lateral canthus, away from the cornea, for 5 minutes with the eyes closed. Schirmer scores were recorded in millimeters of wetting after 5 minutes. TCR was determined based on the rate at which the color of the 0.5% fluorescein dye faded on the Schirmer test strip, and was graded as 1, 1/2, 1/4, 1/8, 1/16, 1/32, 1/64, 1/128, or 1/256; this value was represented in logarithmic form.^[13,26,27]^

Double vital staining method using 1% preservative-free fluorescein (Alcon, Fort Worth, TX) and 1% preservative-free lissamine green (Leiter's Pharmacy, San Jose, CA) dye solutions were used for the staining.^[27]^ Corneal staining scores were obtained by multiplying the area score (0–3) by the density score (0–3).^[28]^ Conjunctival staining scores were determined using the SICCA OSS.^[18]^ Each region was given a score from 0 to 3 based on staining of both the nasal and temporal conjunctivae, and total conjunctival staining scores (6 points maximum) were calculated.^[13]^

### 2.3 Laboratory profile and focus score measurement

Laboratory profiles, including anti-nuclear antibody (ANA), RF levels, erythrocyte sedimentation rate (ESR), and C-reactive protein (CRP) levels were measured at the time of first visit. Autoantibody levels against SS-A/Ro and SS-B/La were determined by enzyme-linked immunosorbent assay. Focus score was investigated in patients of groups 1 and 2, who had SS. Focus score was measured, by a pathologist, as the number of mononuclear cell infiltrates containing at least 50 inflammatory cells in a 4 mm^2^ glandular section in minor salivary gland biopsy.

### 2.4 Statistical analysis

Statistical analysis was performed using Statistical Package for Social Sciences v18.0 for Windows (SPSS Inc., Chicago, IL). The normal distribution for all variables was assessed using the Kolmogorov–Smirnov test. All variables were normally distributed. Data are presented as the mean ± standard deviation. Differences between 3 groups for continuous variables were assessed using analysis of variance (ANOVA) with Bonferroni post hoc analysis. Differences between groups for categorical variables were assessed using the Chi-square test. The student *t* test was used to compare the continuous variables between groups. A *P* value of less than .05 was considered to have statistical significance.

## 3 Results

Group 1 consisted of 71 patients who satisfied the revised 2016 criteria, and group 2 consisted of 20 patients who did not satisfy the newly revised criteria. Fifty-five patients who were diagnosed with non-SS-DE were placed in group 3. The mean age was 52.1 ± 14.6 years in group 1, 53.9 ± 12.0 years in group 2, and 54.2 ± 12.6 years in group 3. Of the 146 total patients, only 3 patients (4.2%) in group 1, 1 (5.0%) in group 2, and 3 (5.4%) in group 3 were males.

**Figure 1:**
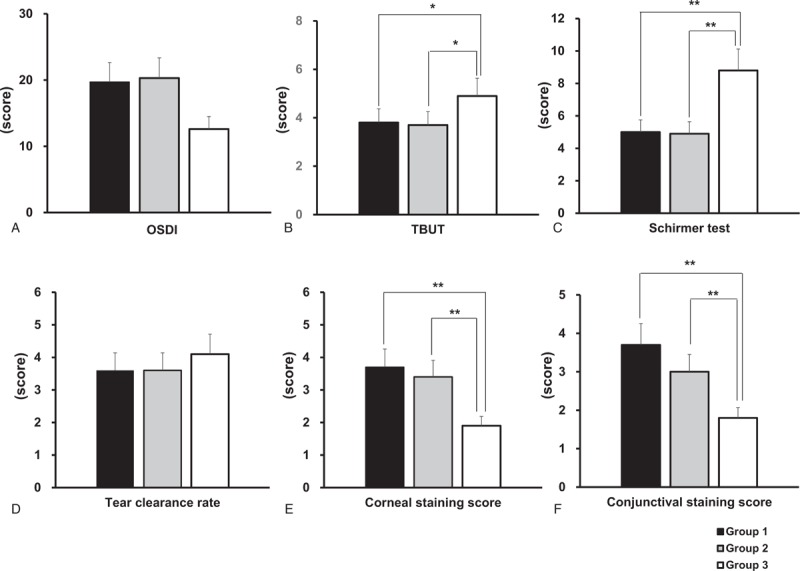
Comparisons of dry eye disease parameters betwee 3 groups; (A) OSDI, (B) TBUT, (C) Schirmer score, (D) tear clearance rate, (E) corneal staining score and (F) conjunctival staining score (^∗^*P* <.05, ^∗∗^*P* <.01). OSDI = ocular surface disease index, TBUT = tear break up time.

Figure 1 shows a comparison of DE disease parameters—OSDI, TBUT, Schirmer score, TCR, corneal staining, and conjunctival staining scores—between the 3 groups. OSDI, indicating the clinical symptoms, was 53.6 ± 19.7 in group 1, 55.3 ± 20.3 in group 2, and 58.2 ± 12.6 in group 3. There were no significant differences between the groups (all *P* >.05) (Fig. 1A). TBUT was 3.8 ± 1.1 in group 1, 3.7 ± 1.5 in group 2, and 4.9 ± 1.6 in group 3 (*P* =.74, group 1 vs 2; *P* = .03, group 1 vs 3; *P* = .03, group 2 vs 3) (Fig. 1B). Schirmer score was 5.0 ± 1.3, 4.9 ± 1.4, and 8.8 ± 2.9 in group 1, 2, and 3, respectively (*P* = .90, group 1 vs group 2; *P* <.01 in both groups 1 and 2 vs group 3) (Fig. 1C). TCR in groups 1, 2, and 3 was 3.6 ± 0.6, 3.6 ± 0.6, and 4.1 ± 0.6, respectively, and there were no significant differences between the groups (all *P* >.05) (Fig. 1D). Corneal and conjunctival staining scores were 3.7 ± 2.4 and 3.7 ± 1.5 in group 1, 3.4 ± 2.8 and 3.0 ± 1.1 in group 2, and 1.9 ± 2.5 and 1.8 ± 1.0 in group 3. Both staining scores were more severe in groups 1 and 2 compared to group 3 (all *P* <.01). However, there were no significant differences in the staining scores between groups 1 and 2 (*P* = .59 and *P* = .10) (Fig. 1E and Fig. 1F).

**Figure 2:**
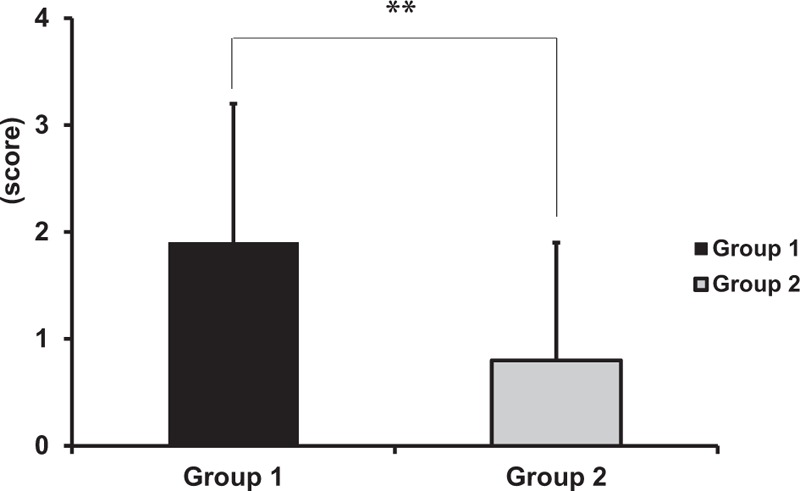
Comparison of focus score between groups 1 and 2 according to satisfaction of the 2016 classification criteria for primary Sjögren's syndrome (^∗∗^*P* <.01).

Table 1 shows laboratory findings according to group. There were no differences between groups 1 and 2 with respect to ANA, RF, ESR, and CRP, but both groups showed higher levels compared to group 3 (all *P* <.01). Only 2 patients (10.0%) in group 2 had anti-SSA antibodies, whereas 61 patients (85.9%) in group 1 had anti-SSA antibodies (*P* <.01). However, there were no significant differences in terms of anti-SSB antibodies between groups 1 and 2 (Table 1). The focus score, which shows the level of lymphocytic infiltration of the salivary gland, was significantly lower in group 2 (0.8 ± 1.1) than in group 1 (1.9 ± 1.3; *P* <.01) (Fig. 2).

**Table 1:**
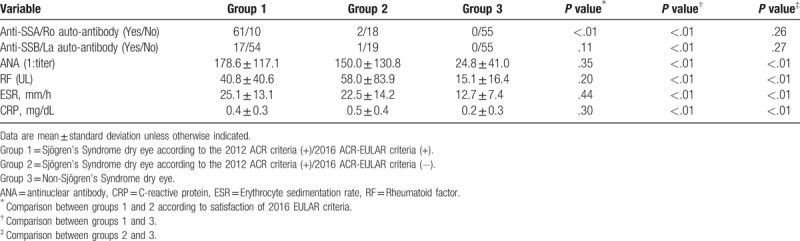
Comparisons of laboratory findings according to groups.

Table 2 shows the details of the classification criteria for primary SS in the 20 patients of group 2. Three patients (15.0%) who were positive for ANA/RF, negative for anti-SSA antibody, and had an OSS of 3 to 4 points did not meet the 2016 ACR classification criteria, despite satisfaction of the focus score. One patient (5.0%) who was positive for anti-SSA antibody, negative for the focus score, and had an OSS of 3 to 4 points was included in group 2. The remaining patients (16 cases, 80.0%) were positive for ANA/RF, but negative for focus score and anti-SSA antibodies, regardless of OSS and Schirmer score.

**Table 2:**
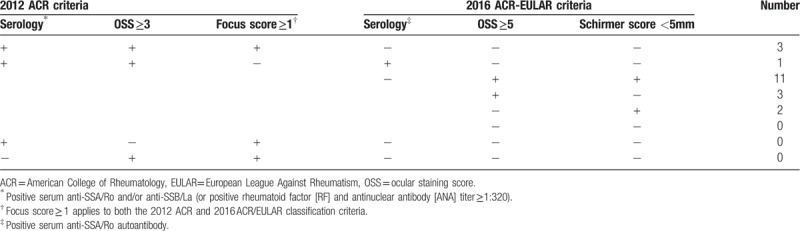
Details of the classification criteria for primary Sjögren's syndrome in patients who satisfied the 2012 ACR criteria, but not the revised 2016 ACR-EULAR criteria (n = 20).

## 4 Discussion

The mechanism of aqueous tear deficiency in DE is known to be increased tear osmolarity and desiccation of the surface epithelium.^[2,29]^ Hyperosmolar tear film and desiccation stress lead to overexpression of inflammatory cytokines (e.g., interleukin (IL)-1β, IL-6, tumor necrosis factor-α, interferon-γ,), chemokines, and matrix metalloproteinases.^[12,30–33]^ and infiltration of CD4 + T cells. These changes cause apoptosis of corneal and lacrimal epithelial cells and a decrease in the density of conjunctival goblet cells, resulting in a vicious cycle of severe DE.^[2,34–36]^ In ocular SS, complex autoimmune responses which are characterized by mononuclear cell infiltration and the presence of autoantibodies like anti-SSA/Ro and anti-SSB/La predominantly occur in the lacrimal gland, with resulting glandular atrophy. The focal infiltration consists mainly of T cells. It has been known that Th17 cells, as well as Th1 cells, increased in the ocular tissues of SS, whereas regulatory T cells were suppressed. Tear production is reduced due to apoptosis of acinar cells by autoimmune response of the lacrimal glands of SS patients.^[2,30]^

SS-related DE has been reported to be associated with more severe clinical manifestations than non-SS DE in many studies.^[2,7,10–12]^ Goto et al^[10]^ showed that tear evaporation rates were significantly higher in SS patients compared with non-SS patients, along with fluorescein/Rose Bengal staining scores. Symptom scores and staining grades were higher, whereas TBUT and tear secretion value were lower in SS-DE than in non-SS-DE.^[7,12]^ In the present study, although no significant difference was found in OSDI score, tear film and ocular surface parameters including TBUT, Schirmer score, and corneal/conjunctival staining scores were worse in groups of SS-DE compared with non-SS DE.

The newly developed 2016 ACR-EULAR criteria were designed to combine features from both the 2012 ACR and 2002 American-European Consensus Group (AECG) criteria for early detection of SS.^[19,20]^ From a rheumatologist's perspective, OSS and lip biopsy are invasive and may be difficult to perform in an outpatient setting. The aim was to achieve this goal by adding Schirmer test and unstimulated salivary flow to the revised 2016 ACR-EULAR criteria. The OSS threshold increased due to the higher specificity. In addition, ANA and RF were excluded as they were considered too nonspecific to be confirmatory for SS.^[19,20]^

Contrary to our expectations, in this study, there were no significant differences in ocular surface parameters, including TBUT, Schirmer score, TCR and corneal/conjunctival staining score, and laboratory findings including ANA, RF, ESR, and CRP between the SS-DE groups according to satisfaction with 2016 revised criteria for SS (group 1 and 2), except for the presence of anti-SSA autoantibodies. In Table 2, most of the patients with pre-existing SS in the group 2, who did not satisfy the 2016 ACR/EULAR criteria, did not meet the focus score outlined in the 2012 criteria. This result corresponds to the result shown in Fig. 2, that a significant difference was observed in focus score between both the SS groups. Taken together, our results show that the difference between both SS groups may be due to the presence of anti-SSA autoantibodies and the focus score, and not due to ocular surface condition.

Recently, DE treatment was set by a stepwise and multidisciplinary approach in the 2017 International Dry Eye WorkShop (DEWS)-II report.^[14,37]^ The ultimate goal of DE management is to restore ocular surface homeostasis and disrupt the vicious cycle of disease by removing the cause.^[14,38]^ DEWS-II report also mentioned that SS-DE is characterized by more severe ocular surface findings than non-SS-DE. SS-DE still requires more aggressive treatment to improve the ocular surface, such as increased potency and concentration of topical steroids and cyclosporine A, autologous serum, and punctal plugs.^[6,14–17]^ For systemic treatment, 50 mg pilocarpine per oral can be used in SS, although conflicting results have been reported.^[1,6,17,39,40]^

In summary, there was no difference in the ocular surface parameters and laboratory findings, except the presence of anti-SSA autoantibodies and the focus score, between the 2 groups with pre-existing SS. Our results suggest that there is no need to change the direction of treatment of DE in patients with pre-existing SS who did not meet the revised 2016 ACR-EULAR criteria. This study has some limitations. Patients in our study were recruited at a single tertiary center, and may not be representative of the characteristics of general DE patients. Multicenter, larger-scale studies are needed to resolve these issues. Additionally, recently commercialized tear analysis tools for non-invasive keratography break-up time, inflammatory parameters, inflammatory mediators, and tear osmolarity could help to identify the severity of DE in patients with SS. It may be helpful to consider newly developed concepts such as tear film oriented diagnosis. Nevertheless, this study was the first to evaluate the characteristics of DE associated with SS according to the different classification criteria for primary SS. This study may be helpful in suggesting directions for DE treatment in patients with SS.

## Author contributions

**Conceptualization:** Kyung Chul Yoon.

**Data curation:** Hyeon Jeong Yoon, Won Choi.

**Formal analysis:** Hyeon Jeong Yoon.

**Funding acquisition:** Kyung Chul Yoon.

**Investigation:** Hyeon Jeong Yoon, Shin-Seok Lee.

**Methodology:** Won Choi, Jee Myung Yang, Yong Sok Ji.

**Writing – original draft:** Hyeon Jeong Yoon.

**Writing – review & editing:** Kyung Chul Yoon.
